# Combination of platelet-to-lymphocyte ratio and D-dimer for the identification of cardiogenic cerebral embolism in non-valvular atrial fibrillation

**DOI:** 10.3389/fneur.2023.1069261

**Published:** 2023-02-08

**Authors:** Yachen Shi, Chenhao Xuan, Wei Ji, Feng Wang, Jin Huang, Lei Li, Hui Wang, Jingyu Deng, Junfei Shao, Kefei Chen, Xuqiang Mao, Qinghua Xu, Yiping You, Guangjun Xi

**Affiliations:** ^1^Department of Neurology, The Affiliated Wuxi People's Hospital of Nanjing Medical University, Wuxi, China; ^2^Department of Interventional Neurology, The Affiliated Wuxi People's Hospital of Nanjing Medical University, Wuxi, China; ^3^Department of Functional Neurology, The Affiliated Wuxi People's Hospital of Nanjing Medical University, Wuxi, China; ^4^Department of Critical Care Medicine, The Affiliated Wuxi People's Hospital of Nanjing Medical University, Wuxi, China; ^5^Department of Neurosurgery, The Affiliated Wuxi People's Hospital of Nanjing Medical University, Wuxi, China

**Keywords:** non-valvular atrial fibrillation, cardiogenic cerebral embolism, platelet-to-lymphocyte ratio, D-dimer, least absolute shrinkage and selection operator

## Abstract

**Background:**

Non-valvular atrial fibrillation (NVAF) is the most common cause of cardiogenic cerebral embolism (CCE). However, the underlying mechanism between cerebral embolism and NVAF is indefinite, and there is no effective and convenient biomarker to identify potential risk of CCE in patients with NVAF in clinic. The present study aims to identify risk factors for interpreting the potential association of CCE with NVAF and providing valuable biomarkers to predict the risk of CCE for NVAF patients.

**Methods:**

641 NVAF patients diagnosed with CCE and 284 NVAF patients without any history of stroke were recruited in the present study. Clinical data including demographic characteristics, medical history, and clinical assessments, were recorded. Meanwhile, Blood cell counts, lipid profiles, high-sensitivity C-reactive protein, and coagulation function-related indicators were measured. Least absolute shrinkage and selection operator (LASSO) regression analysis was utilized to build a composite indicator model based on the blood risk factors.

**Results:**

(1) CCE patients had significantly increased neutrophil-to-lymphocyte ratio, platelet-to-lymphocyte ratio (PLR), and D-dimer levels as compared with patients in the NVAF group, and these three indicators can distinguish CCE patients from ones in the NVAF group with an area under the curve (AUC) value of over 0.750, respectively. (2) Using the LASSO model, a composite indicator, i.e., the risk score, was determined based on PLR and D-dimer and displayed differential power for distinguishing CCE patients from NVAF patients with an AUC value of over 0.934. (3) The risk score was positively correlated with the National Institutes of Health Stroke Scale and CHADS2 scores in CCE patients. (4) There was a significant association between the change value of the risk score and the recurrence time of stroke in initial CCE patients.

**Conclusions:**

The PLR and D-dimer represent an aggravated process of inflammation and thrombosis in the occurrence of CCE after NVAF. The combination of these two risk factors can contribute to identifying the risk of CCE for patients with NVAF with an accuracy of 93.4%, and the greater in change of composite indicator, the shorter in the recurrence of CCE for NVAF patients.

## Introduction

Approximately 15–20% of ischemic strokes in clinic are caused by cardiogenic embolism, namely, cardiogenic cerebral embolism (CCE), which is one of the main subtype in the Acute Stroke Treatment classification (1, 2) and displays high mortality, disability, and rate of relapse (3). Atrial fibrillation, especially non-valvular atrial fibrillation (NVAF), is the most common cardiac arrhythmia associated with an increase in risk of CCE, and the autopsy study indicates that about 56% of the cardiac thrombus in stroke patients were caused by NVAF (4). Hemodynamic disorders, inflammatory response, and abnormal activation of coagulation are crucial mechanisms in the pathogenesis of thrombosis, and the continuous exacerbation of these pathological processes in NVAF will increase the risk of thrombus detachment, which can further embolize the intracranial artery and lead to CCE (5). Furthermore, many previous studies also revealed that oral anticoagulants including warfarin and non-vitamin K antagonist anticoagulants, were associated with lower risks of ischemic stroke in NVAF patients (6–8). However, the underlying mechanism between cerebral embolism and NVAF is indefinite. Although the utilization of CHADS2 and CHA2DS2-VASc score contribute to predicting the risk of stroke in NVAF patients (9, 10), there is no dependable biomarker to identify potential risk of CCE for NVAF patients until now.

Blood-related indicators can be obtained conveniently and provide valuable information in the clinical practice. A potential biological interplay between white blood cell counts and platelet may be involve in the arterial thrombosis (11, 12), and several specific combinations, e.g., lymphocyte-to-monocyte ratio (LMR), neutrophil-to-lymphocyte ratio (NLR), and platelet-to-lymphocyte ratio (PLR), as important inflammation markers, are associated with the neurological deterioration and long-term prognosis in CCE patients with NVAF (13–15). Furthermore, D-dimer can reflect fibrin formation and degradation, and elevated levels of it is a potential factor for increased risk of ischemic stroke in NVAF patients (16, 17). Other coagulation activation markers (e.g., fibrinogen, prothrombin) showed abnormally changed levels in acute ischemic stroke with NVAF (18). However, it is unclear which risk factors can predict the risk of CCE in NVAF patients, and the potential relationship of these risk factors with the occurrence of CCE in NVAF.

In the present study, we aimed to identify risk factors for interpreting the potential association of CCE with NVAF. Subsequently, a composite indicator will be built for predicting the risk of CCE for NVAF patients. Furthermore, the full-scale clinical assessments of the composite indicator will be investigated further based on the severe of stroke, anticoagulants, and recurrence of CCE.

## Materials and methods

### Patient selection

Nine hundred and twenty-five patients with NVAF were recruited in the present study from the Wuxi People's Hospital. Among them, 641 NVAF patients with CCE (named as the CCE group) and 284 NVAF patients who did not have any history of stroke (named as the control group). This study protocol was approved by the Ethics Committee of the Affiliated Wuxi People's Hospital of Nanjing Medical University (approval number: KY22070) and followed the tenets of the Declaration of Helsinki. All patients or their legal guardians signed written informed consent.

The inclusion criteria were as follows: (1) age 50–85 years old; and (2) history of NVAF, or confirmed NVAF during admission according to an electrocardiogram or dynamic electrocardiogram. CCE patients may be caused by emboli in the heart and meet the following criteria: (I) at least one associated cardiac source of emboli is determined; (II) acute ischemic stroke, confirmed by brain computed tomography / magnetic resonance imaging, are similar to large artery atherosclerosis ischemic stroke; and (III) stroke in more than one vascular area or systemic embolism evidence supports the diagnosis of CCE (4, 19). Meanwhile, NVAF patients showed matched age, sex, and medical history, however, they did not have any history of stroke, including transient ischemic attack. Furthermore, the exclusion criteria included: (1) a severe consciousness disorder; (2) valvular heart disease or congenital heart disease; (3) serious systemic diseases, such as infection, lung failure, heart failure (III–IV stage), liver and kidney dysfunction, or tumor; or (4) other diseases, including serious endocrine disease, severe trauma, peripheral vascular disease, or mental disorders.

### Medical history and data collection

Data on demographics and clinical data regarding the index event were extracted. Basic information of age, sex, smoking, and comorbidities (e.g., hypertension, diabetes mellitus, coronary disease, stroke, and cardiac failure) was recorded. Additionally, for all patients, the CHADS2 scores were measured, and the National Institutes of Health Stroke Scale (NIHSS) scores only were measured in CCE patients for assessing the stroke severity.

In addition, anticoagulation therapy of each patient was also recorded in the CCE group. Warfarin, dabigatran, and rivaroxaban, as the primary anticoagulants were used, and patients using the single anticoagulant were included in the present study.

### Blood sample collection and test

Peripheral venous blood was collected into an EDTA-coated tube, a vacutainer tube (without anticoagulant), and a sodiumcitrate anticoagulation tube between 7:00 and 8:00 a.m. after overnight fasting.

Blood cell counts, lipid profiles, high-sensitivity C-reactive protein (hs-CRP), and coagulation function-related indicators, e.g., international normalized ratio (INR), fibrinogen, D-dimer, were detected. Furthermore, some specific blood indexes were further calculated based on blood cell counts, e.g., LMR, NLR, and PLR.

### Statistical analysis

SPSS 16.0 software (SPSS, Inc., Chicago, IL) was used to conduct statistical analyses. The Kolmogorov-Smirnov test was used to determine the normal distribution of the data, and the Levene's homogeneity of variance test was also utilized. Continuous data is presented as the mean ± standard deviation if variables followed a normal distribution, and variables with non-normal distribution are presented as medians [interquartile range (IQR)]. The independent-sample *t*-test or Mann–Whitney *U* test was used for univariate analyses of continuous variables. A chi-squared test was used for the categorical variables. Paired *t*-test was used for analyzing the changed level of variate between initial and second hospital records. Multivariate analysis was performed using the logistic regression model, and the odds radio (OR) and 95% confidence interval (CI) were obtained. The receiver operating characteristic (ROC) curve analysis was performed to assess the diagnostic power of relevant indicators. Youden's index (20) was used to determine optimal values (cutoff value), sensitivity and specificity. Spearman correlation analysis was used to find associations between the risk score and NIHSS or CHADS2 scores. Kruskal–Wallis test was utilized to evaluate the association of the risk score with different anticoagulation therapy. Statistically significant result requires a *P*-value of 0.05 or less.

Furthermore, to assess the potential risk of CCE for NVAF patients, least absolute shrinkage and selection operator (LASSO) regression analysis was performed using R (4.2.1) software to construct a risk score model based on the valuable blood indicators and a composite indicator (i.e., the risk score) would be identified (21, 22).

## Results

### Characteristics of participants

As shown in Table 1, with the univariate analysis, the CCE group showed significantly increased white blood cell count (WBC), monocyte count, neutrophil count, NLR, PLR, hs-CRP, triglyceride (TG), INR, fibrinogen, and D-dimer levels, and significantly reduced lymphocyte count and NLR levels compared with the NVAF group (all *P* < 0.05). Additionally, there was no significant difference in other clinical characteristics and the use of anticoagulant between the two groups (all *P* > 0.05). These blood risk factors with significant difference may have a potential to identify the risk of CCE for NVAF patients.

**Table 1:** Comparison of clinical characteristics between CCE patients with NVAF and single NVAF patients.

	**CCE (*N* = 641)**	**NVAF (*N* = 284)**	***P***
Age (years)	73.00 (67.00–79.00)	71.00 (64.00–77.75)	0.080^†^
Sex, male	328 (51.17%)	158 (55.63%)	0.118^‡^
Hypertension	442 (68.95%)	186 (65.49%)	0.168^‡^
Diabetes mellitus	165 (25.74%)	61 (21.48%)	0.095^‡^
Coronary disease	183 (28.55%)	92 (32.39%)	0.135^‡^
Previous stroke	199 (31.05%)	0 (0.00%)	< 0.001^‡^
Cardiac failure	169 (26.37%)	78 (27.46%)	0.393^‡^
Smoking	128 (19.97%)	61 (21.48%)	0.329^‡^
NIHSS score	2.00 (2.00–5.00)	–	–
CHADS2 score	2.00 (2.00–3.00)	2.00 (1.00–2.00)	< 0.001^†^
WBC ( × 10^9^/L)	6.97 (5.60–8.65)	5.61 (4.74–6.68)	< 0.001^†^
Lymphocyte count ( × 10^9^/L)	1.47 (1.09–1.81)	1.75 (1.32–2.26)	< 0.001^†^
Monocyte count ( × 10^9^/L)	0.56 (0.43–0.71)	0.50 (0.40–0.61)	< 0.001^†^
Neutrophil count ( × 10^9^/L)	4.56 (3.42–6.23)	3.30 (2.58–4.14)	< 0.001^†^
Platelet count ( × 10^9^/L)	174.00 (138.50–211.00)	176.00 (143.25–215.75)	0.244^†^
LMR	2.56 (1.87–3.53)	3.61 (2.76–4.52)	< 0.001^†^
NLR	3.09 (2.08–5.06)	1.82 (1.42–2.45)	< 0.001^†^
PLR	120.45 (90.56–160.66)	52.67 (38.27–69.02)	< 0.001^†^
Hs-CRP (mg/L)	3.10 (0.90–9.78)	0.6 (0.50–1.60)	< 0.001^†^
TC (mmol/L)	4.20 ± 1.12	4.15 ± 0.97	0.499^§^
TG (mmol/L)	1.14 (0.86–1.61)	1.29 (0.96–1.81)	0.002^†^
LDL-c (mmol/L)	2.42 ± 0.88	2.32 ± 0.74	0.111^§^
HDL-c (mmol/L)	1.03 ± 0.26	1.04 ± 0.25	0.882^§^
INR	1.08 (1.02–1.17)	1.10 (1.03–1.21)	0.008^†^
Fibrinogen (g/L)	2.77 (2.34–3.37)	2.58 (2.27–3.01)	< 0.001^†^
D-dimer (ug/L)	302.00 (170.00–649.50)	98.50 (55.00–152.50)	< 0.001^†^
Anticoagulant, used number	221 (34.48%)	105 (36.97%)	0.255^‡^

Data is presented as mean ± standard deviation/number (percentage of total) / median (25 percentile to 75 percentile).

CCE, cardiogenic cerebral embolism; NVAF, non-valvular atrial fibrillation; NIHSS, National Institutes of Health Stroke Scale; TC, total cholesterol; WBC, white blood cell count; LMR, lymphocyte-to-monocyte ratio; NLR, neutrophil-to-lymphocyte ratio; PLR, platelet-to-lymphocyte ratio; Hs-CRP, high-sensitivity C-reactive protein; TG, triglyceride; LDL-c, low-density lipoprotein cholesterol; HDL-c, high-density lipoprotein cholesterol; INR, international normalized ratio.

† Mann–Whitney U-test.

‡ Chi-squared test.

§ Independent-sample t-test.

To comprehensively evaluate the association between these blood risk factors and the occurrence of CCE in NVAF patients and determine the most valuable risk factors, a multivariate analysis followed using logistics analysis was performed, and the results were exhibited in Table 2. The analysis revealed that WBC (OR = 2.049, 95% CI = 1.562–2.689, *P* < 0.001), lymphocyte count (OR = 0.139, 95% CI = 0.039–0.486, *P* = 0.002), neutrophil count (OR = 1.519, 95% CI = 1.074–2.150, *P* = 0.018), NLR (OR = 0.503, 95% CI = 0.348–0.726, *P* < 0.001), PLR (OR = 1.066, 95% CI = 1.055–1.077, *P* < 0.001), and D-dimer (OR = 1.005, 95% CI = 1.003–1.006, *P* < 0.001) were related to CCE onset, and these six risk factors exhibited a greater potential to predict the occurrence of CCE in NVAF.

**Table 2:** Logistics analysis for the association between clinical features with significant difference in univariate analyses and the occurrence of CCE.

	**OR**	**95%CI**	***P***
WBC	2.049	1.562–2.689	< 0.001
Lymphocyte count	0.139	0.039–0.486	0.002
Monocyte count	7.498	0.255–220.544	0.243
Neutrophil count	1.519	1.074–2.150	0.018
LMR	1.497	0.953–2.354	0.080
NLR	0.503	0.348–0.726	< 0.001
PLR	1.066	1.055–1.077	< 0.001
Hs-CRP	1.072	0.995–1.155	0.069
TG	0.929	0.784–1.100	0.391
INR	0.992	0.558–1.763	0.977
Fibrinogen	1.059	0.719–1.560	0.772
D-dimer	1.005	1.003–1.006	< 0.001

CCE, cardiogenic cerebral embolism; WBC, white blood cell count; LMR, lymphocyte-to-monocyte ratio; NLR, neutrophil-to-lymphocyte ratio; PLR, platelet-to-lymphocyte ratio; Hs-CRP, high-sensitivity C-reactive protein; TG, triglyceride; INR, international normalized ratio.

### Determination of composite indicator

**Figure 1:**
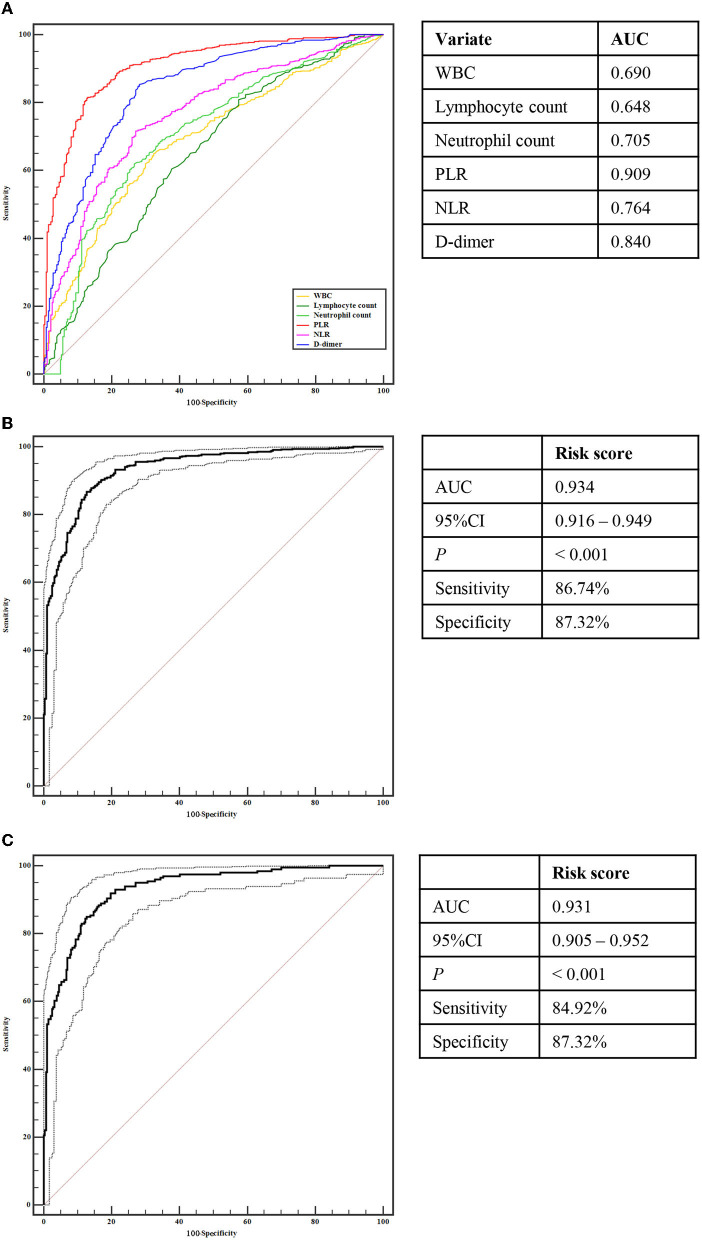
Receiver operating characteristic curve analyses. **(A)** Analyses of WBC, Lymphocyte count, Neutrophil count, PLR, NLR, and D-dimer for identifying CCE patients with from NVAF patients without stoke. **(B)** Analyses of the risk score for identifying CCE patients with from NVAF patients without stoke. **(C)** Analyses of the risk score for identifying initial CCE patients with from NVAF patients without stoke. CCE, cardiogenic cerebral embolism; NVAF, non-valvular atrial fibrillation; WBC, white blood cell count; PLR, platelet-to-lymphocyte ratio; NLR, neutrophil-to-lymphocyte ratio.

Using ROC analysis, the differential power of these six risk factors for CCE are displayed in Figure 1A. Among them, PLR, D-dimer, and NLR could accurately distinguish CCE patients from those in the NVAF group with an area under the curve (AUC) > 0.750, which showed the greater accuracy to identify the risk of CCE.

**Figure 2:**
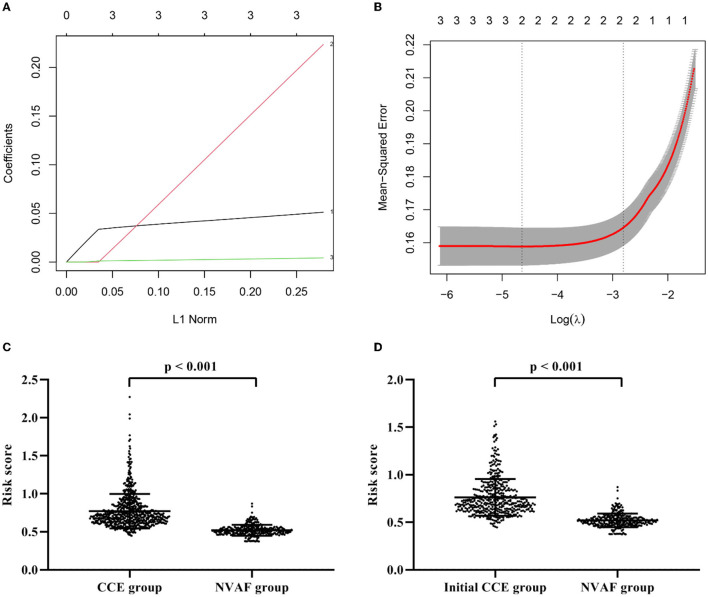
Construction of a composite indicator using least absolute shrinkage and selection operator model. **(A)** The coefficients of each variate in the model (1: D-dimer; 2: PLR; 3: NLR). **(B)** The mean-squared error of model. **(C)** The significant difference in the risk score between CCE patients and single NVAF patients. **(D)** The significant difference in the risk score between initial CCE patients and single NVAF patients. CCE, cardiogenic cerebral embolism; NVAF, non-valvular atrial fibrillation; PLR, platelet-to-lymphocyte ratio; NLR, neutrophil-to-lymphocyte ratio.

Compared with the single indicator, a composite indicator may display more clinical value. To identify a comprehensive risk index to predict the occurrence of CCE in NVAF patients, we built a composite indicator (i.e., the risk score) based on the PLR, D-dimer, and NLR. The risk score of the LASSO model was calculated as follows: *risk score* = *the level of PLR* × *2.508250e*^−03^ + *the level of D-dimer* × *9.086806e*^−05^ + *0.3645818* (Figures 2A, B). However, the NLR was eliminated due to the coefficient of NLR in this model is zero (Figure 2A). Figure 2C showed the significant difference in the risk score between CCE and NVAF groups (*P* < 0.001), and the risk score could differentiate CCE patients from patients in the NVAF group with an AUC of 0.934 (sensitivity = 86.74%, specificity = 87.32%, cutoff value = 0.74; Figure 1B). Additionally, there were 442 NVAF patients with the initial ischemic stroke in the CCE group, thus, we further assess the diagnostic performance of the risk score to identify the initial CCE. The significant difference in the risk score between the initial CCE group and the NVAF group was observed (*P* < 0.001; Figure 2D), and the ROC curve analysis indicated that the risk score could differentiate initial CCE patients from patients in the NVAF group with similarly higher accuracy (AUC = 0.931, sensitivity = 84.92%, specificity = 87.32%, cutoff value = 0.74; Figure 1C). The new composite indicator, the risk score, showed an outstanding clinical value for identifying the risk of CCE in NVAF.

### Association of the risk score with stroke in CCE

**Figure 3:**
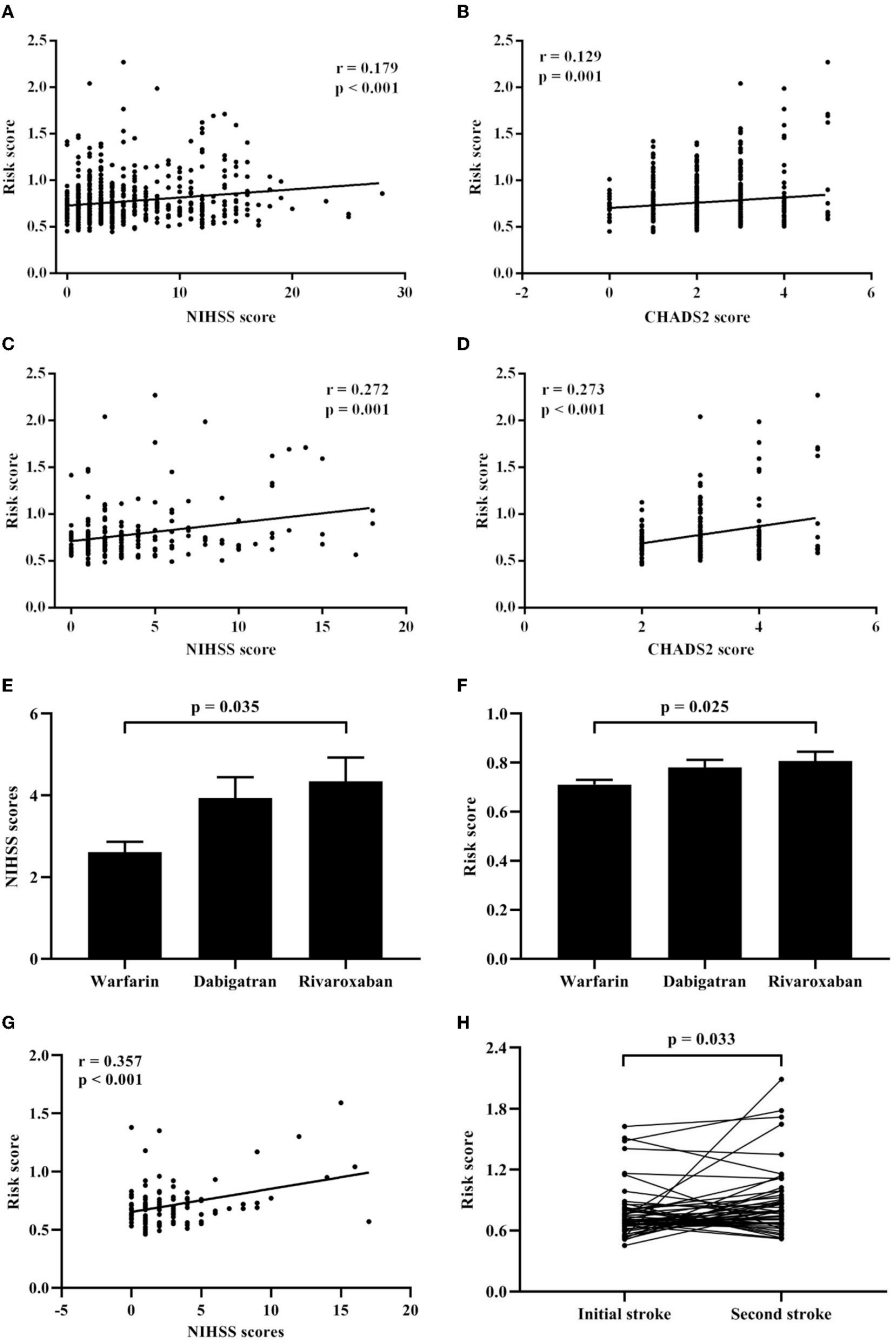
Associations of the risk score with clinical features in CCE patients. **(A)** Correlation analysis between the risk score and the NIHSS scores in CCE patients. **(B)** Correlation analysis between the risk score and the CHADS2 scores in CCE patients. **(C)** Correlation analysis between the risk score and the NIHSS scores in initial CCE patients. **(D)** Correlation analysis between the risk score and the CHADS2 scores in initial CCE patients. **(E)** Difference of NIHSS scores among warfarin, dabigatran, and rivaroxaban groups in CCE patients. **(F)** Difference of the risk score among warfarin, dabigatran, and rivaroxaban groups in CCE patients. **(G)** Correlation analysis between the risk score and the NIHSS scores in CCE patients with the treatment of warfarin. **(H)** Difference analysis of the risk score between the first and second hospitalization in CCE patients.

Spearman's rank correlation analysis revealed that the risk score positively correlated with the NIHSS scores (*r* = 0.179, *p* < 0.001; Figure 3A) and the CHADS2 scores (*r* = 0.129, *p* = 0.001; Figure 3B) in the CCE group. Besides, in CCE patients with the initial stroke, the risk score showed also positive correlations with the NIHSS scores (*r* = 0.272, *p* = 0.001; Figure 3C) and the CHADS2 scores (*r* = 0.273, *p* < 0.001; Figure 3D).

Furthermore, in the CCE group, 221 patients received a kind of anticoagulation therapy before being hospitalized (warfarin: 103 ones; dabigatran: 74 ones; rivaroxaban: 44 ones). Results showed that there were significant differences in the NIHSS scores and the risk score among three treatment groups (NIHSS score: H = 6.624, *P* = 0.036; risk score: *H* = 8.341, *P* = 0.015), and patients with warfarin exhibited significantly lower NIHSS scores and risk score than those with rivaroxaban (Figures 3E, F). Meanwhile, a significant correlation between the NIHSS scores and the risk score was found in patients with warfarin (*r* = 0.375, *p* < 0.001; Figure 3G), however, the correlation between the NIHSS scores and the risk score was non-significant in other two treatment groups.

### Association of the risk score with recurrent stroke in initial CCE

For initial CCE patients, 49 patients with the recurrent stroke were re-hospitalized and recorded in the current study. Among them, 21 patients showed the recurrent stroke within a year ( ≤ 12 months) and 25 ones had the second stroke after more than 12 months. Compared with the risk score during the first hospitalization, 49 patients showed significant increased higher risk score when they had stroke again (Figure 3H). Δ risk score represents the change value of the risk score for each patients, which was calculated by “last risk score—initial risk score.” Regression analysis indicated that the Δ risk score was significantly associated with the interval of recurrence of stroke in initial CCE patients, controlling age, sex, smoking, comorbidities, initial NIHSS score, initial risk score and anticoagulation therapy (Table 3).

**Table 3:** Logistics analysis for the association of clinical features with the interval of recurrence of CCE.

	**OR**	**95%CI**	***P***
Age	1.070	0.970–1.182	0.177
Sex	1.020	0.151–6.865	0.984
Hypertension	0.368	0.049–2.773	0.332
Diabetes mellitus	2.242	0.393–12.800	0.364
Coronary disease	2.106	0.344–12.893	0.420
Cardiac failure	0.800	0.137–4.681	0.805
Smoking	0.132	0.013–1.369	0.090
Initial NIHSS score	0.959	0.829–1.109	0.570
Initial risk score	0.389	0.012–12.520	0.594
Anticoagulation therapy^§^	0.227	0.044–1.173	0.077
Δ risk score	0.018	0.001–0.575	0.023

CCE, cardiogenic cerebral embolism; NIHSS, National Institutes of Health Stroke Scale.

§ Anticoagulation therapy indicated whether the use of anticoagulation at the second hospitalization, rather than which anticoagulation was used.

## Discussion

The present study main found that (1) significantly changed levels of WBC, lymphocyte count, neutrophil count, NLR, PLR, and D-dimer were associated with the occurrence of CCE in NVAF patients, and among these risk factors, PLR and D-dimer showed the closer association with CCE; (2) using the LASSO model, a composite indicator, the risk score, was determined based on levels of PLR and D-dimer and displayed greatly differential power for distinguishing CCE patients from patients in the NVAF group with an accuracy of 93.4%; (3) the risk score can reflect the severe of stroke in CCE patients; (4) warfarin may decrease the risk score to result in relatively mild stroke symptoms for NVAF patients with the ischemic stroke; (5) in initial CCE patients, the greater the increase in risk score, the shorter the relapse interval of stroke. In the present study, the most valuable risk factors were identified by a progressive analysis, i.e., univariate analysis, multivariate analysis, ROC analysis and mathematical model. Meanwhile, correlation and regression analyses were further used to assess the clinical value of the risk score. Taken together, with a large sample size, the risk score may contribute to identifying NVAF patients with a high risk of CCE and predicting the recurrence of CCE. Furthermore, the present study provided a convenient approach, a blood test of PLR and D-dimer, to identify accurately the risk of CCE.

In consistent with the previous studies (13), levels of PLR were significantly increased in the CCE group as compared with the NVAF group. In recent years, high PLR levels have emerged as a novel laboratory marker for reflecting increased level of inflammation and thrombosis (23), and the significant associations of PLR with the severity of neurological impairment and prognosis in ischemic stroke were observed in previous studies (24, 25). In addition, as a widely used marker of hypercoagulable states and thrombosis, D-dimer levels were significant elevated in CCE patients in the present study, which is consistent with the findings of previous studies (26, 27). Recently, Yuan et al. revealed that high D-dimer levels may be related with the high risk of ischemic stroke rather than hemorrhagic stroke (28), and Choi et al. indicated that patients with the higher D-dimer levels displayed a higher probability of recurrent embolic ischemic stroke (29). Therefore, increased levels of PLR and D-dimer may be important clinical indicators for the occurrence of CCE in NVAF patients.

In the present study, the determination of “risk score” using the LASSO algorithm based on the levels of PLR and D-dimer, can provide a comprehensive risk marker to identify the embolic ischemic stroke in NVAF. Although single indicator (e.g., PLR, NLR, or D-dimer) showed acceptably distinguishing power, the risk score is recommended for optimal differential diagnosis of CCE patients from patients in the NVAF group, and either for total CCE or for initial CCE, the risk score exhibited the great generalizability and applicability. Importantly, in the present study, significant correlations of risk score with the NIHSS and CHADS2 scores in CCE patients suggested that higher risk score reflected the more severe stroke symptoms and the higher risk of stroke for NVAF. Meanwhile, there was a significant correlation between lower NIHSS scores and lower risk score in CCE patient with the treatment of warfarin, which indicated that CCE patients exhibited the milder stroke symptoms probably due to use of warfarin in NVAF to reduce the level of risk score. Furthermore, our study also found that by controlling several covariates, for those initial CCE patient with the recurrent stroke, the second risk score was significantly higher than the first risk score, and the higher change value of the risk score, the shorter the interval of recurrence of second stroke after the initial CCE. Hence, the risk score may be a valuable and novel clinical indicator to recognize the occurrence and recurrence of CCE for NVAF patients.

Although we adequately estimated the potential clinical significance of the risk score for the NVAF-related CCE, there were still several limitations in the present study. (1) Some underlying confounding factors may exist in the present analysis, e.g., different pharmacotherapy for comorbidities and differential lifestyles for each participant, which may have an effect on the blood test results and the present findings. A rigorous study design is essential to control these confounders and to verify the present findings. (2) The present study is a single-center study. In the future, we will perform a multi-center study to confirm the present results, which contributes to assess the repeatability and generalization of the risk score. Furthermore, different types of stroke (e.g., large artery atherosclerosis stroke) and atrial fibrillation (e.g., valvular atrial fibrillation) should be considered to assess the specificity of clinical application of the risk score for identifying CCE in NVAF patients. (3) Some previous studies indicated that non-vitamin K antagonist oral anticoagulants may have better effect for stroke prevention than warfarin in NVAF (8, 30, 31), which is inconsistent with our findings. Less participants with the use of rivaroxaban are included in the present CCE patients, which may be the main cause to result in the current findings. (4) There are less records of the follow-up for CCE patients in the present study, which limits the ability to fully evaluate the predictive value of the risk score for the recurrence of CCE. In our subsequent study, the long-term prediction effect of the risk score on the second occurrence of CCE will be the main focus.

## Conclusion

The present study demonstrated that elevated PLR and D-dimer levels showed the greater value for identifying the occurrence of CCE in NVAF patients, which supported that the aggravation of inflammation and thrombosis was associated with the CCE after NVAF. Furthermore, the composite indicator, the risk score, was determined using the LASSO regression analysis based on the levels PLR and D-dimer, which exhibited better performance with an accuracy of 93.4% to distinguish CCE patients from patients in the NVAF group than the single PLR or D-dimer. In addition, the clinical associations of stroke severe and treatment-related change with the risk score can further facilitate the application of this new indicator for CCE in NVAF. Furthermore, the greater in change of the risk score, the shorter in the recurrence of CCE for NVAF patients, which suggested that this new indicator can contribute to predicting the recurrence of stroke for NVAF patients.

## Data availability statement

The original contributions presented in the study are included in the article/supplementary material, further inquiries can be directed to the corresponding authors.

## Ethics statement

The studies involving human participants were reviewed and approved by Ethics Committee of the Affiliated Wuxi People's Hospital of Nanjing Medical University. The patients/participants provided their written informed consent to participate in this study. Written informed consent was obtained from the individual(s) for the publication of any potentially identifiable images or data included in this article.

## Author contributions

YS, WJ, and GX drafted the manuscript and contributed to the discussion. CX, FW, JH, LL, HW, JD, XM, and QX collected the data and analyzed the data. YS and CX analyzed the data. JS and KC contributed to the discussion. YY and GX designed the study and contributed to the discussion and revised the manuscript. All authors contributed to the article and approved the submitted version.
